# Efficacy and safety of anlotinib in the treatment of brain metastases from non-small cell lung cancer: a systematic review and meta-analysis

**DOI:** 10.3389/fphar.2025.1607255

**Published:** 2025-09-25

**Authors:** Chunyue Fang, Congbo Yue, Junzhu Wu, Tianliang Yao, Jin Wen, Wei Dai, Qionghui Yang, Ruixiang Chen, Qinghuan Li, Yuanyuan Zhong

**Affiliations:** ^1^ College of Pharmacy, Dali University, Dali, Yunnan, China; ^2^ Department of Pharmacy, The Third People's Hospital of Yunnan Province, Kunming, Yunnan, China; ^3^ Department of Clinical Laboratory, Peking University People's Hospital, Qingdao, Shandong, China; ^4^ Department of Clinical Laboratory, Women and Children's Hospital, Qingdao University, Qingdao, Shandong, China; ^5^ Department of Control Science and Engineering, College of Electronic and Information Engineering, Tongji University, Shanghai, China

**Keywords:** anlotinib, non-small cell lung cancer, brain metastases, meta-analysis, efficacy, safety, randomized controlled trial, cohort study

## Abstract

**Background:**

The presence of brain metastases is a significant factor contributing to the failure of lung cancer therapies. This study aims to systematically assess the efficacy and safety of anlotinib in the treatment of brain metastases resulting from non-small cell lung cancer (NSCLC).

**Methods:**

A comprehensive literature search was conducted across multiple databases, including PubMed, EMBASE, Cochrane Library, Web of Science, CNKI, Wan fang, and VIP databases. This search encompassed the period from the establishment of the databases up until November 2024. The Cochrane risk of bias tool was employed to assess the quality of the randomized controlled trials (RCTs) included in the study, while the quality of the cohort studies was evaluated using the Newcastle-Ottawa Scale (NOS) score. The Grading of Recommendations Assessment, Development and Evaluation (GRADE) system was used to evaluate the quality of evidence. The meta-analysis was conducted using the RevMan 5.3 software.

**Results:**

A total of 18 studies (9 RCTs and 9 retrospective cohort studies; n = 1,480) were included in the analysis. The results demonstrated that, compared to the control group, anlotinib significantly prolonged intracranial progression-free survival (IPFS)[3 studies; HR = 0.52, 95% CI (0.36–0.75), P = 0.0004, I^2^ = 0%, fixed-effect model] and improved overall survival (OS) [3 studies; HR = 0.69, 95% CI (0.54–0.88), P = 0.0003, I^2^ = 0%, fixed-effect model]. Furthermore, anlotinib increased the objective response rate (ORR) [15 studies; RR = 1.55, 95% CI (1.30–1.84), P < 0.00001, I^2^ = 58%, random-effects model] and disease control rate (DCR) [13 studies; RR = 1.33, 95% CI (1.23–1.44), P < 0.00001, I^2^ = 29%, fixed-effect model]. Regarding safety outcomes, the anlotinib group showed a significantly reduced risk of nausea-vomiting [7 studies; RR = 0.52, 95% CI (0.29–0.92), P = 0.02, I^2^ = 0%, fixed-effect model], while no significant differences were observed in other adverse reactions.

**Conclusion:**

Anlotinib may prolong IPFS and OS in patients with brain metastases from NSCLC, and may improve ORR and DCR. In terms of safety, may reduce the risk of nausea-vomiting. The risk of bias in some of the included studies was unclear. The quality of OS and DCR was moderate, while that of IPFS, ORR and nausea-vomiting was low.

**Systematic Review Registration:**

https://www.crd.york.ac.uk/PROSPERO/view/CRD42025632195, identifier [CRD42025632195].

## 1 Introduction

Lung cancer is one of the common malignant tumours with high morbidity and mortality worldwide, the most commonly diagnosed cancer in 2022 is lung cancer with nearly 2.5 million new cases, accounting for 12.4% of all cancers globally, and it is also the leading cause of cancer deaths with an estimated 1.8 million deaths, accounting for 18.7% of cancer fatalities (Bray et al., 2024). Non-small cell lung cancer (NSCLC) is the predominant histological subtype, comprising over 80% of lung cancer cases. Additionally, around 20% of NSCLC patients present with brain metastases at initial diagnosis, and approximately 50% develop brain metastases during the course of treatment (Page et al., 2020). The presence of brain metastases is a significant factor contributing to the failure of lung cancer therapies, and the overall efficacy of surgical interventions, radiotherapy, and other treatment modalities remains unsatisfactory.

Anlotinib, a novel oral small-molecule multi-target tyrosine kinase inhibitor (TKI) developed in China, received official approval from the China Food and Drug Administration on May 9, 2018. Current clinical guidelines (Index Medicus Global, 2020) endorse its use as a third-line treatment and beyond for NSCLC, positioning it as a new therapeutic option for lung cancer. The mechanism of action of anlotinib primarily involves the effective inhibition of various targets, including the vascular endothelial growth factor receptor (VEGFR), platelet-derived growth factor receptor (PDGFR), fibroblast growth factor receptor (FGFR), and c-Kit, which collectively contribute to tumor growth inhibition and the suppression of tumor-associated neovascularization (Shuai and Yan, 2023). Tumor growth and survival are contingent upon the acquisition of nutrients and oxygen through newly formed blood vessels, which facilitate sustained growth and metastasis. Consequently, obstructing the formation of tumor neovascularization and the associated nutrient supply represents a viable strategy for effective tumor inhibition and treatment (Gu et al., 2024). Anlotinib is effective in treating non-small cell lung cancer (Zhou et al., 2024), small cell lung cancer (Liying and Fang, 2024; Xianming et al., 2024; Yun et al., 2024), breast cancer (Fan et al., 2024; Weili et al., 2024), among other types. While existing studies have indicated the efficacy of anlotinib in treating NSCLC with brain metastases (Lian et al., 2021; Liu et al., 2022; Guohong et al., 2023; Gu et al., 2024; Yong et al., 2024), a comprehensive evaluation of its efficacy and safety in this specific patient population has yet to be conducted. Therefore, this paper seeks to systematically assess the efficacy and safety of anlotinib in the management of brain metastases in patients with non-small cell lung cancer, thereby providing evidence-based medical insights to inform clinical decision-making.

## 2 Materials and methods

### 2.1 Research registration

This systematic review and meta-analysis were conducted in accordance with the Cochrane Handbook for Systematic Reviews of Interventions (Version 6.5, 2024), and the Preferred Reporting Items for Systematic Reviews and Meta-Analyses (PRISMA) 2020 statement (Supplementary Material S1). The research initiative has been registered with the Prospective Registry for Systematic Reviews (PROSPERO) under the registration number CRD42025632195.

### 2.2 Literature search and methodology

A systematic search was performed utilizing the PubMed, EMBASE, Cochrane Library, Web of Science, CNKI, Wan fang, and VIP databases. The search terms employed included Anlotinib, AL3818, non-small cell lung cancer, non-small cell carcinoma, non-small cell lung tumor, NSCLC, and brain metastasis. The search was conducted from the construction of the database to November 2024, using a combination of free word and subject word searches. The complete search strategies for each database can be found in Supplementary Material S2.

### 2.3 Inclusion and exclusion criteria

Inclusion Criteria: (1) Patients diagnosed with non-small cell lung cancer (NSCLC) exhibiting brain metastases; (2) Inclusion of randomized controlled trials (RCTs) or retrospective cohort studies; (3) Availability of complete and accurate research data; (4) The control group received either chemotherapy or a placebo, while the experimental group was administered anlotinib in conjunction with the control treatment; (5) Outcome measures included objective response rate (ORR), disease control rate (DCR), intracranial progression-free survival (IPFS), overall survival (OS), and adverse effects. IPFS is defined as the time from the start of treatment to intracranial progression or death; OS is based on the time from the start of treatment to death or the last follow-up time; ORR = (CR + PR)/total number × 100%; DCR = (CR + PR + SD)/total number × 100%.

Exclusion Criteria: (1) Repeated literature; (2) Literature for which full text cannot be obtained; (3) Conference abstracts; (4) Literature from which key data cannot be extracted, converted or obtained; (5) Low-quality literature:Sample size is too small (n < 20); incorrect data analysis methods; numerous grammatical errors and typos; logical confusion.

### 2.4 Literature selection and data extraction

The documents retrieved from PubMed, EMBASE, Cochrane Library, Web of Science, CNKI, Wan fang, and VIP were imported into Endnote, and the duplicate documents were removed using the function of review. Two researchers simultaneously read the literature, first excluded the non-compliant literature by reading the title and abstract, and then read through the remaining literature and extracted the compliant literature according to the literature inclusion and exclusion criteria. In the course of the screening procedure, should there be a discrepancy in the assessments of two researchers, a third researcher is consulted to resolve the differences. Some key information about the literature that was eventually included was extracted, such as first author, year of publication, number of patient cases, interventions, outcome indicators, and other information.

### 2.5 Literature quality evaluation

For the literature quality evaluation of RCTs, risk of bias was assessed using the Co-chrane Systematic Evaluation’s Manual 5.2.0 recommended bias assessment tool Rob.2.0, with risk of bias graded as high risk of bias, unknown risk of bias and low risk of bias. The evaluation was conducted independently by 2 researchers, and when there was disagreement on the assessment, it was assessed by a third researcher. Literature quality assessment for retrospective cohort studies was scored according to the Newcastle-Ottawa Scale (NOS), which consists of three parts: selection of study subjects, comparability between groups, and outcome measures, with a total score of 9. The higher the score, the better the quality.

The analysis of publication bias was conducted using Stata 18.0 software. For outcome indicators (IPFS and OS) with fewer than 10 included studies, we did not assess publication bias through funnel plots or statistical tests, as these methods are insufficiently effective in small sample sizes. The quality of evidence is evaluated using the GRADE system. GRADE is an internationally recognized system for grading the quality of evidence and the strength of recommendations, widely used in systematic reviews, clinical guidelines, and other fields. Evidence quality is categorized into four levels: high, moderate, low, and very low. The evaluation criteria include five aspects: risk of bias, inconsistency, indirectness, imprecision, and publication bias.

### 2.6 Statistical methods

Statistical Analysis Application: Data analysis was performed utilizing Review Manager 5.3 software. The ORR, DCR, and metrics related to adverse reactions were evaluated through the calculation of relative risk (RR) alongside a 95% confidence interval (95% CI). IPFS and OS were analyzed using the hazard ratio (HR) and a 95% confidence interval (95% CI). The selection of random-effects or fixed-effects models was based on a comprehensive assessment of clinical, methodological, and statistical heterogeneity. For clinical heterogeneity, it should be determined based on the characteristics of the study population, interventions, definitions of outcome indicators and measurement methods; for methodological heterogeneity, it should be determined based on the type of study design and the risk of bias (ROB assessment results, NOS scores); and for statistical heterogeneity, the assessment of heterogeneity was conducted via the Q test (α = 0.1), with quantitative analysis executed using the I^2^ statistic. Z-test, with P ≤ 0.05 being considered statistically different. Publication bias was assessed using a funnel plot in conjunction with Egger’s regression test via Stata 18.0 software. A P ≤ 0.05 was considered statistically significant. The trim-and-fill method was applied to evaluate and adjust for potential bias in the results.

## 3 Results

### 3.1 Outcomes of the literature review

**FIGURE 1:**
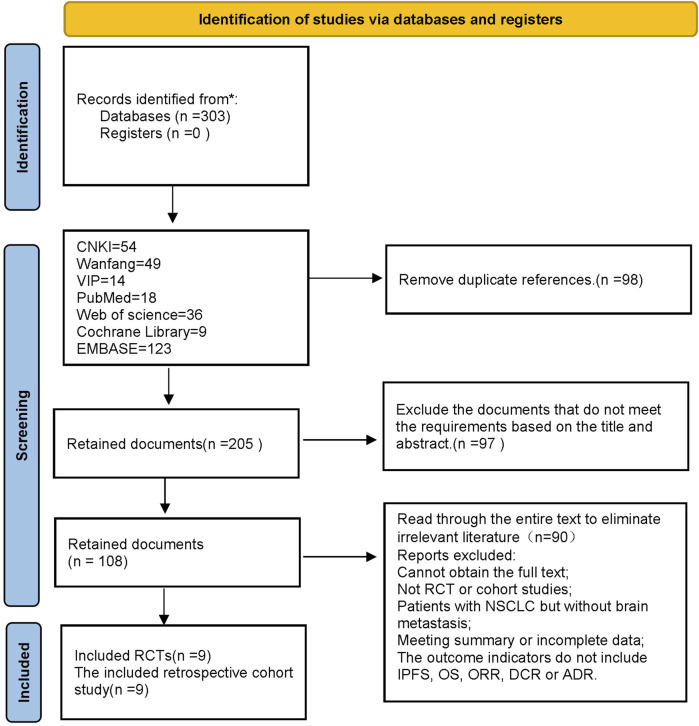
Literature screening flowchart.

In this study, a total of 303 pieces of related literature were retrieved, and 205 pieces were left after eliminating duplicates, 97 pieces of non-compliant literature were excluded by reading the titles and abstracts of the literature, and the remaining 108 pieces of literature were read through the whole text, and the final pieces of literature were selected based on the inclusion and exclusion criteria, and 18 pieces of literature were finally included in the study with a total of 1,480 patients, and the flowchart of the literature screening process is shown in Figure 1. The 18 pieces of literature contained 9 RCTs and 9 retrospective cohort studies, and the basic characteristics of the literature are shown in Table 1, 2.

**TABLE 1:** Basic characteristics of RCTs.

Study	Treatment group	Control group	Treatment group interventions	Control group interventions	Outcome indicator
Hongju et al. (2023)	44	44	Anlotinib + Radiotherapy	Radiotherapy	response rate, adverse events, death rate, quality of life
Guiying (2023)	18	18	Anlotinib + Radiotherapy	Radiotherapy	response rate, survival rate, Adverse event rates, quality of life
Kun (2022)	49	49	Anlotinib + Radiotherapy	Radiotherapy	treatment efficacy, serological index, adverse reaction
Qingsong (2022)	20	20	Anlotinib + Hypofractionated Radiotherapy	Hypofractionated Radiotherapy	treatment efficacy (ORR and DCR),Incidence of adverse reactions
Yi (2022)	50	50	Anlotinib + Stereotactic radiotherapy	Stereotactic radiotherapy	Serum tumour markers, quality of life, adverse reaction
Guoqing et al. (2024)	29	32	Anlotinib + Icotinib Hydrochloride	Icotinib Hydrochloride	Long-term near-term outcome, Intracranial lesions efficacy, safety
Erping et al. (2020)	26	26	Anlotinib + Radiotherapy	Radiotherapy	serum level, Incidence of adverse reactions,DCR,efficiency
Xingzhi et al. (2020)	20	20	Anlotinib + Radiotherapy	Radiotherapy	treatment efficacy, adverse reaction, survival rate
Jiang et al. (2020)	294	143	Anlotinib	placebo-controlled study	PFS,OS,Intracranial objective response rate,DCR

**TABLE 2:** Basic characteristics of cohort studies.

Study	Treatment group	Control group	Treatment group interventions	Control group interventions	Outcome indicator
Jie et al. (2023)	21	21	Anlotinib + whole-brain radiotherapy	whole-brain radiotherapy	Intracranial objective response rate, Intracranial disease control rate, IPFS, OS, adverse reaction
Ming (2021)	20	20	Anlotinib + radiotherapy alone	radiotherapy alone	CR, PR, SD, PD, ORR, DCR
Yuxia et al. (2022)	21	25	Anlotinib + Stereotactic radiotherapy	Stereotactic radiotherapy	Efficacy of treatment of intracranial lesions, CRN incidence, IPFS
Kai (2022)	23	35	Anlotinib + Radiotherapy	Radiotherapy	Treatment efficiency, Serum CEA carcinoembryonic antigen, adverse reaction
Dongying et al. (2022)	30	30	Anlotinib + whole-brain radiotherapy	whole-brain radiotherapy	treatment efficacy, toxicological reaction, IPFS, OS
Manhua et al. (2022)	30	33	Anlotinib + Hstereotactic radiotherapy	Hypofractionated stereotactic radiotherapy	treatment efficacy, Incidence of adverse reactions
He et al. (2021)	28	45	Anlotinib + Cranial Radiotherapy	Cranial Radiotherapy	OS, Extracranial PFS, PFS
Kong et al. (2023)	34	42	Anlotinib + Wholebrain radiotherapy	Wholebrain radiotherapy	IPFS , OS, safety
Liu et al. (2025)	21	43	Anlotinib + Wholebrain radiotherapy	Wholebrain radiotherapy	IPFS , OS, toxic

### 3.2 Quality assessment of the included literature

**FIGURE 2:**
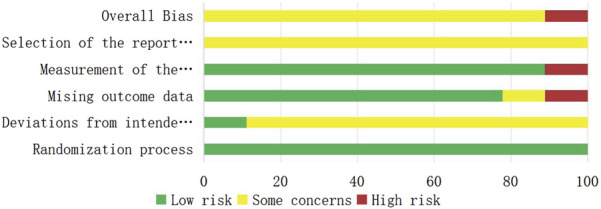
Literature quality evaluation chart.

**FIGURE 3:**
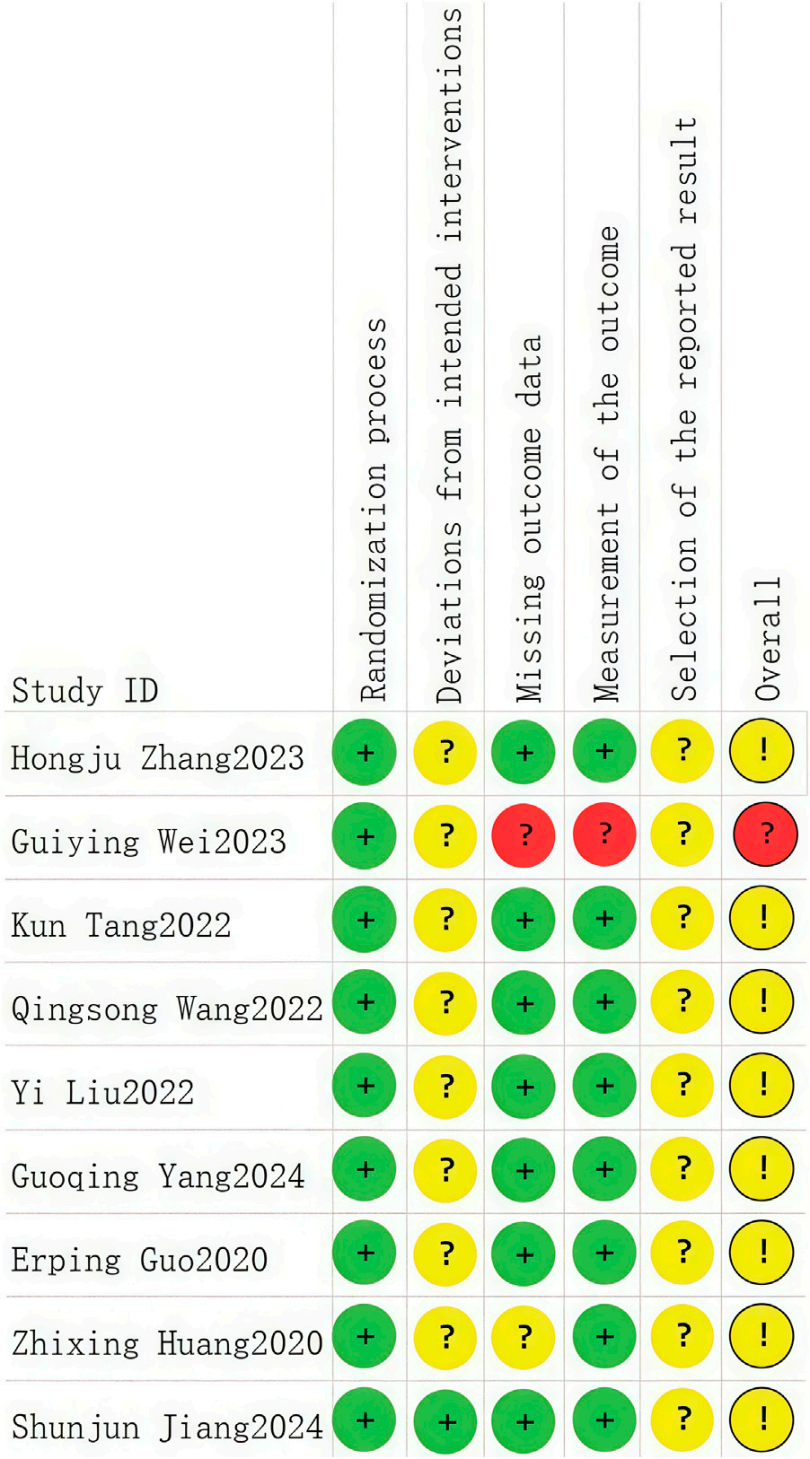
Literature quality evaluation chart.

A total of nine RCTs (Erping et al., 2020; Jiang et al., 2020; Xingzhi et al., 2020; Kun, 2022; Qingsong, 2022; Yi, 2022; Guiying, 2023; Hongju et al., 2023; Guoqing et al., 2024) were analyzed in accordance with the bias risk assessment criteria established by the Cochrane Collaboration. Among these studies, three (Qingsong, 2022; Yi, 2022; Guoqing et al., 2024) utilized the random number table method, one study (Hongju et al., 2023) employed computer-generated random assignment, while five studies (Erping et al., 2020; Jiang et al., 2020; Xingzhi et al., 2020; Kun, 2022; Guiying, 2023) did not specify the method of random sequence generation. Among the included literature, one study (Xingzhi et al., 2020) used single blindness and one study (Jiang et al., 2020) used double blindness. All nine studies (Erping et al., 2020; Jiang et al., 2020; Xingzhi et al., 2020; Kun, 2022; Qingsong, 2022; Yi, 2022; Guiying, 2023; Hongju et al., 2023; Guoqing et al., 2024) did not adequately address the potential for bias due to selective reporting of results, leading to an assessment of unclear risk. The findings from the quality evaluation are illustrated in Figures 2, 3, the “Overall Bias” in the figure encompasses five aspects: selection of the reported result, measurement of the outcome, missing outcome data, deviations from the intended intervention, and the randomization process. In Figure 2, “Overall Bias” is a summary of the risk of bias across the 9 RCTs, while in Figure 3, “Overall Bias” is a summary of the individual risk of bias for each of the 9 RCTs. Additionally, the nine retrospective cohort studies included (He et al., 2021; Ming, 2021; Dongying et al., 2022; Kai, 2022; Manhua et al., 2022; Yuxia et al., 2022; Jie et al., 2023; Kong et al., 2023; Liu et al., 2025) were assessed using the Newcastle-Ottawa Scale (NOS), achieving a total score of 9. The scoring outcomes for each of these studies are detailed in Table 3.

**TABLE 3:** NOS scale rating scale.

Author	Selection/score	Comparability/score	Exposure/score	Total/score
Jie et al. (2023)	4	2	3	9
Ming (2021)	4	0	1	5
Yuxia et al. (2022)	4	2	2	8
Kai (2022)	4	0	2	6
Dongying et al. (2022)	4	2	2	8
Manhua et al. (2022)	4	2	2	8
He et al. (2021)	4	2	2	8
Kong et al. (2023)	4	2	3	9
Liu et al. (2025)	4	0	2	6

### 3.3 Results of the meta-analysis

#### 3.3.1 Intracranial progression-free survival (IPFS)

**FIGURE 4:**
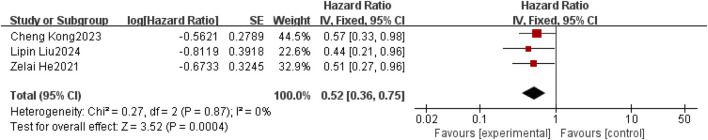
Intracranial progression-free survivalin of three cohort studies.

Three studies (He et al., 2021; Kong et al., 2023; Liu et al., 2025) reported HR values for IPFS. Considering the relatively low clinical and methodological heterogeneity, with I^2^ = 0% and P = 0.87, the data were pooled using a fixed-effect model. Meta results showed a statistically significant improvement in IPFS in patients with non-small cell lung cancer brain metastases with the use of anlotinib compared to patients with non-small cell brain metastases without the use of anlotinib [HR = 0.52, 95% CI (0.36,0.75), P = 0.0004], as shown in Figure 4.

#### 3.3.2 Overall survival (OS)

**FIGURE 5:**
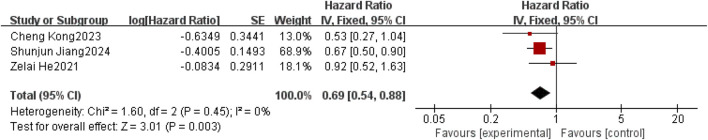
Overall survival of two cohort studies and one randomized controlled trial.

Overall survival was reported in three (Jiang et al., 2020; He et al., 2021; Kong et al., 2023). Considering the relatively low clinical and methodological heterogeneity, along with I^2^ = 0%,P = 0.45, a fixed-effect model was used to aggregate the data. Meta-results showed a statistically significant improvement in OS in patients with brain metastases from non-small-cell lung cancer with the use of anlotinib compared to patients with non-small-cell brain metastases without the use of anlotinib [HR = 0.69, 95% CI (0.54,0.88), P = 0.0003], as shown in Figure 5.

#### 3.3.3 Objective response rate (ORR)

Fifteen studies (Erping et al., 2020; Xingzhi et al., 2020; He et al., 2021; Ming, 2021; Dongying et al., 2022; Kai, 2022; Kun, 2022; Manhua et al., 2022; Qingsong, 2022; Yuxia et al., 2022; Guiying, 2023; Hongju et al., 2023; Jie et al., 2023; Guoqing et al., 2024; Liu et al., 2025) reported objective remission rates between patients with non-small cell lung cancer brain metastases with and without the use of anlotinib. Taking into account the clinical and methodological heterogeneity, as well as the relatively high I^2^ value (58%), P = 0.003, a random effects model was used to summarize the data.The Meta-analysis results showed that compared with non-small cell lung cancer patients with brain metastases who did not use anlotinib, the use of anlotinib improved the ORR of these patients [RR = 1.55, 95% CI (1.30, 1.84), P < 0.00001].

**FIGURE 6:**
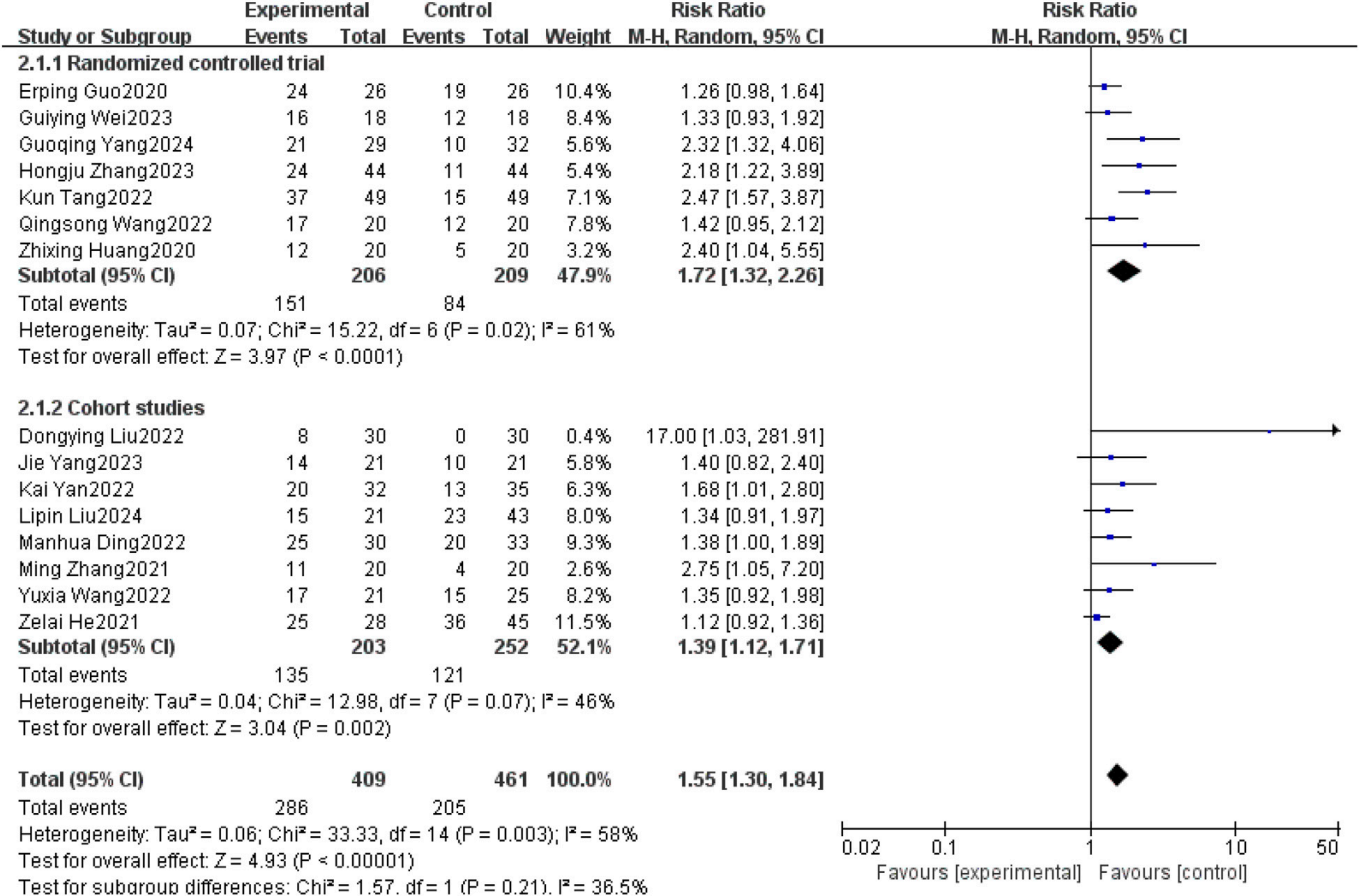
Objective response rate.

Further subgroup analysis by study type revealed that in the RCT subgroup [RR = 1.72, 95% CI (1.32, 2.26), P < 0.0001], the ORR of patients using anlotinib was significantly higher than that of patients not using anlotinib; in the cohort study subgroup [RR = 1.39, 95% CI (1.12, 1.71), P = 0.002], a similar finding was observed that using anlotinib could significantly improve the ORR of patients. The results of the above subgroup analysis were consistent with the overall analysis conclusion, both supporting the improvement effect of anlotinib on the ORR of patients with brain metastases from non-small cell lung cancer, as shown in Figure 6.

#### 3.3.4 Disease control rate (DCR)

Thirteen studies (Erping et al., 2020; Xingzhi et al., 2020; Ming, 2021; Dongying et al., 2022; Kai, 2022; Kun, 2022; Manhua et al., 2022; Qingsong, 2022; Yuxia et al., 2022; Hongju et al., 2023; Jie et al., 2023; Guoqing et al., 2024; Liu et al., 2025) reported DCR between patients with brain metastases from non-small cell lung cancer using and without anlotinib. Considering the relatively low heterogeneity in both clinical and methodological aspects, with I^2^ = 29% and P = 0.16, a fixed-effect model was used to summarize the data. The Meta results showed that the use of anlotinib could improve the DCR of non-small cell lung cancer patients with brain metastases compared with non-small cell lung cancer patients without anlotinib, and the difference was statistically significant [RR = 1.33, 95% CI(1.23, 1.44), P < 0.00001].

**FIGURE 7:**
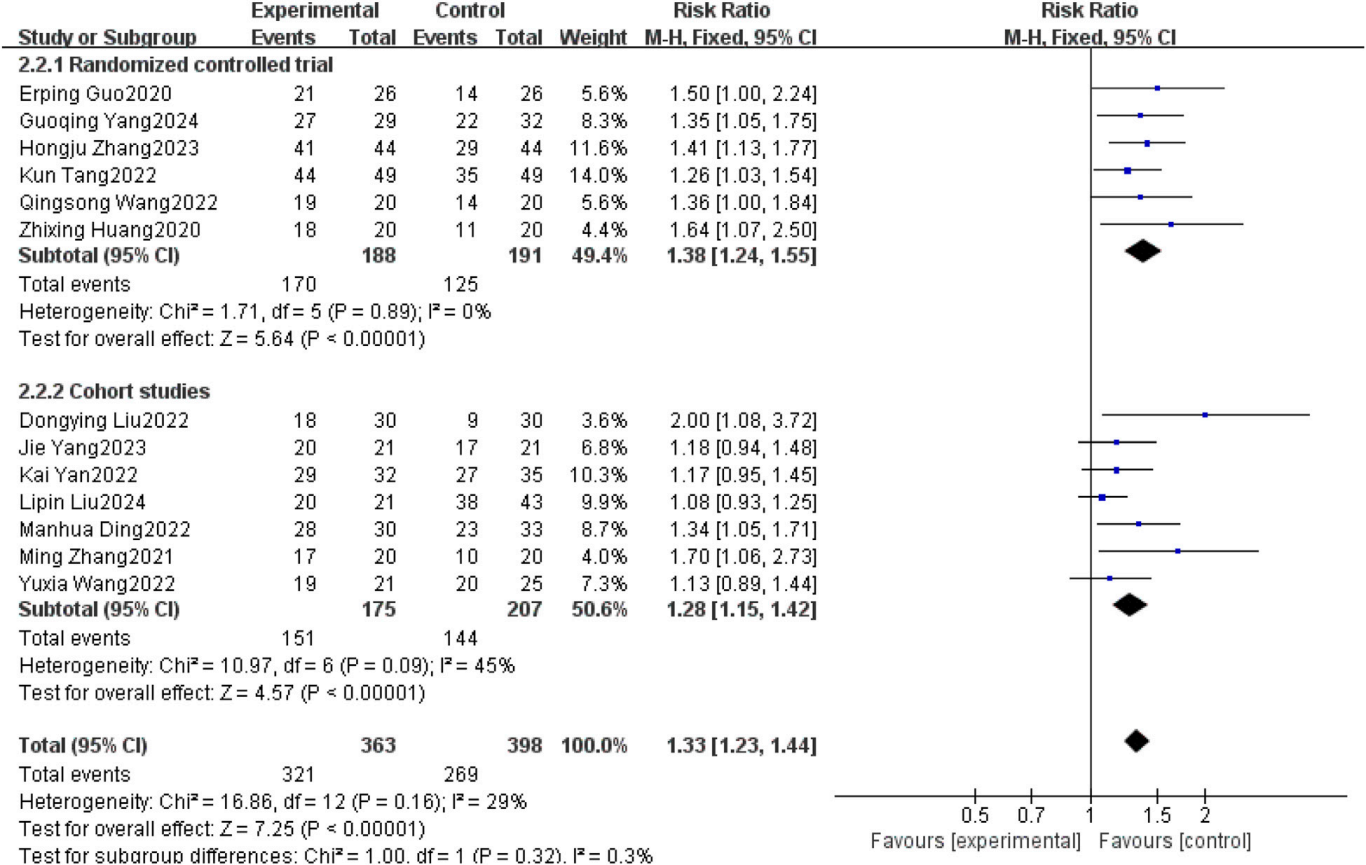
Disease control rates.

Further subgroup analysis by study type revealed that in the RCT subgroup [RR = 1.38, 95% CI (1.24, 1.55), P < 0.00001], the DCR of patients using anlotinib was significantly higher than that of patients not using anlotinib; in the cohort study subgroup [RR = 1.28, 95% CI (1.15, 1.42), P < 0.00001], it was also observed that using anlotinib could significantly improve the DCR of patients. The results of the above subgroup analysis were consistent with the overall analysis conclusion, both supporting the improvement effect of anlotinib on the DCR of patients with brain metastases from non-small cell lung cancer, as shown in Figure 7.

#### 3.3.5 Safety

**FIGURE 8:**
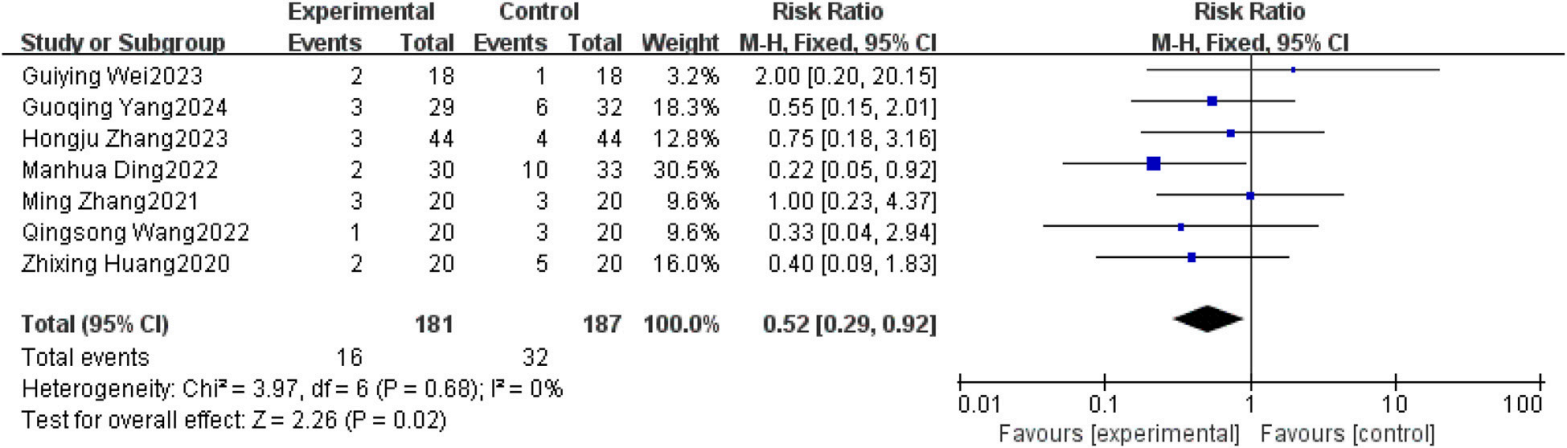
Nausea-vomiting.

Seven studies (Xingzhi et al., 2020; Ming, 2021; Manhua et al., 2022; Qingsong, 2022; Guiying, 2023; Hongju et al., 2023; Guoqing et al., 2024) reported nausea-vomiting in patients with brain metastases from non-small cell lung cancer using and without anlotinib. Considering the relatively low heterogeneity in both clinical and methodological aspects, along with I^2^ = 0% and p = 0.68, a fixed-effect model was used to summarize the data. The Meta results showed that there was a correlation between the use of anlotinib and nausea and vomiting, and the difference was statistically significant [RR = 0.52, 95%CI (0.29, 0.92), P = 0.02], as shown in Figure 8. There was no statistical significance in the incidence of diarrhea, leukopenia, anemia, hypertension, hand-foot syndrome, skin problems, albuminuria and headache between the two groups (P > 0.05). The results are shown in Table 4.

**TABLE 4:** Meta analysis results of various adverse reactions.

Adverse reaction	Number of studies	Heterogeneity		Effect model	RR (95%CI)	P
		P	I^2^ (%)			
constipation	5 (Xingzhi et al., 2020; Ming, 2021; Kai, 2022; Yi, 2022; Liu et al., 2025)	0.74	0%	Fixed-effects model	0.63 [0.31,1.15]	0.12
decreased white blood cells	8 (Xingzhi et al., 2020; Ming, 2021; Kun, 2022; Qingsong, 2022; Guiying, 2023; Hongju et al., 2023; Guoqing et al., 2024; Liu et al., 2025)	0.86	0%	Fixed-effects model	0.73 [0.39,1.40]	0.35
anemia	2 (Qingsong, 2022; Hongju et al., 2023)	0.85	0%	Fixed-effects model	1.67 [0.42,6.68]	0.47
hypertensive	5 (Kai, 2022; Manhua et al., 2022; Yi, 2022; Guoqing et al., 2024; Liu et al., 2025)	0.02	67%	Random-effects models	2.27 [0.44,11.63]	0.33
hand-foot syndrome	3 (Manhua et al., 2022; Qingsong, 2022; Liu et al., 2025)	0.29	19%	Fixed-effects model	2.49 [0.63,9.83	0.19
skin problem	6 (Xingzhi et al., 2020; Ming, 2021; Kai, 2022; Yi, 2022; Guiying, 2023; Guoqing et al., 2024)	0.20	32%	Fixed-effects model	0.82 [0.43,1.57]	0.55
albuminuria	2 (Kai, 2022; Manhua et al., 2022)	0.68	0%	Fixed -effects model	0.35 [0.10,1.24]	0.10
headache	2 (Manhua et al., 2022; Liu et al., 2025)	0.42	0%	Fixed-effects model	5.34 [0.79 [36.27]	0.09

### 3.4 Publication bias analysis

The number of studies included for IPFS and OS was limited (both were 3), which was insufficient to support the creation of a funnel plot. Therefore, a funnel plot was drawn using ORR and DCR as indicators, and a “trim-and-fill” test was conducted.

**FIGURE 9:**
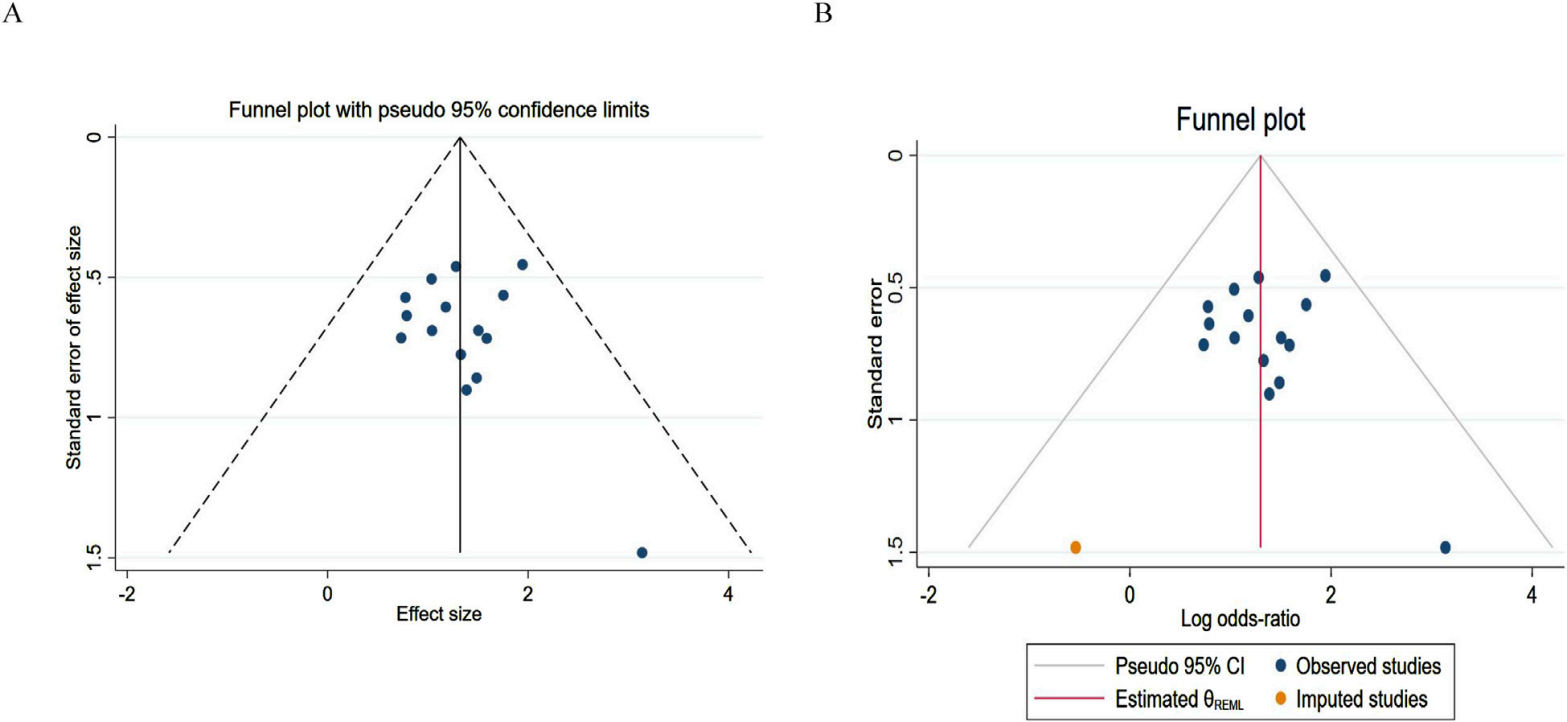
Funnel plot of objective response rate. **(A)** Pre-trim-and-fill; **(B)** Post-trim-and-fill correction.

For the publication bias analysis of ORR, Figure 9A shows a point asymmetry (dense on the left and sparse on the right). After using the trim-and-fill correction and imputing 2 item, the result is shown in Figure 9B. The log odds ratio of the existing studies (Observed) is 1.322, with a 95% confidence interval of [1.002, 1.641]. After including the imputed studies (Observed + Imputed), the log odds ratio is 1.299, with a 95% confidence interval of [0.982, 1.617]. After imputation, the log odds ratio slightly decreased, and the lower limit of the confidence interval decreased while the upper limit contracted. This indicates that the original conclusion (ORR improvement) has a risk of being “overestimated” due to publication bias.

**FIGURE 10:**
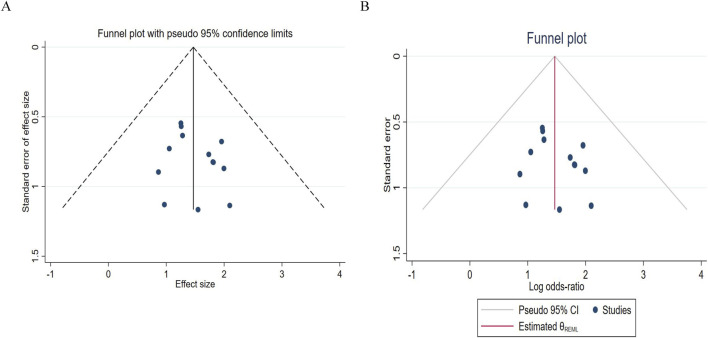
Funnel chart of disease control rates. **(A)** Pre - trim-and-fill; **(B)** Post-trim - and-fill correction.

For the publication bias analysis of DCR, Figure 10A shows that the points are almost symmetrical, with no extreme points deviating from the confidence limits. Figure 10B shows that most of the points are within the interval. This further indicates that the results are stable and there is no serious bias. Before and after imputation, the effect size is completely consistent, with a log odds ratio of 1.468 and a 95% confidence interval of [1.055, 1.881], indicating that publication bias has almost no impact on the DCR conclusion.

### 3.5 GRADE quality of evidence assessment

For the outcome indicators included in the meta-analysis: IPFS, OS, ORR, DCR, and nausea-vomiting, the GRADE evidence quality assessment was conducted. The assessment results showed that OS and DCR were of moderate quality evidence, while IPFS, ORR, and nausea-vomiting were of low quality evidence. The results are shown in Supplementary Material S3.

## 4 Discussion

The results of this study showed that patients with brain metastases from non-small cell lung cancer receiving anlotinib combined therapy may experience prolonged IPFS, and OS, as well as potential benefits in terms of ORR and DCR. The results of the study of Yong et al. (2024) also showed that the combined treatment of anlotinib had good effects, with CR in 1 case (5.9%), PR in 14 cases (82.4%), intracranial objective response rate in 88.2%, and Intracranial disease control rate in 100%. The median IPFS was 10.8 months and the median OS was 20.4 months.

The results of the IPFS study in this study were [HR = 0.52, 95% CI (0.36,0.75), P = 0.0004], and the results of overall survival were [HR = 0.69, 95% CI (0.54,0.88), P = 0.0003], and the differences of both IPFS and overall survival were statistically significant (P < 0.05). The results showed that the improvement of IPFS and OS in patients with non-small cell lung cancer brain metastases by the combination of anlotinib was statistically significant compared with the control group, but further validation is needed because the sample size is too small. The objective remission rate of this study incorporated the findings of 15 (Erping et al., 2020; Xingzhi et al., 2020; He et al., 2021; Ming, 2021; Dongying et al., 2022; Kai, 2022; Kun, 2022; Manhua et al., 2022; Qingsong, 2022; Yuxia et al., 2022; Guiying, 2023; Hongju et al., 2023; Jie et al., 2023; Guoqing et al., 2024; Liu et al., 2025) literature for Meta-analysis [RR = 1.55, 95% CI (1.30, 1.84), P < 0.00001], which showed that the combination of anlotinib significantly improved the objective remission rate of patients compared with the control group. DCR were incorporated from 13 studies in the (Erping et al., 2020; Xingzhi et al., 2020; Ming, 2021; Dongying et al., 2022; Kai, 2022; Kun, 2022; Manhua et al., 2022; Qingsong, 2022; Yuxia et al., 2022; Hongju et al., 2023; Jie et al., 2023; Guoqing et al., 2024; Liu et al., 2025) literature, and Meta-analysis showed [RR = 1.33, 95% CI (1.23, 1.44), P < 0.00001] that the combination of anlotinib significantly improved DCR in patients compared to the control group. In the ORR analysis, I^2^ = 58%, P = 0.003, with I^2^ > 50% indicating moderate heterogeneity. The heterogeneity in ORR may primarily be attributed to the following factors: Firstly, the diversity of treatment regimens (radiotherapy/chemotherapy/targeted therapy vs. anlotinib combined with radiotherapy/chemotherapy/targeted therapy), and differences in radiotherapy modalities such as radiation dose, target volume directly impact the local control efficacy of intracranial tumors, which may lead to fluctuations in ORR. Secondly, differences in baseline patient characteristics including number of metastases, mutation status, prior treatment history. Thirdly, subtle differences in assessment criteria. These factors may collectively contribute to heterogeneity. Future subgroup analyses (e.g., stratified by “type of combined radiotherapy” or “EGFR mutation status”) could further quantify the contribution of each factor to heterogeneity, providing guidance for clinically precise selection of anlotinib combination regimens.

In terms of safety, hypertension, albuminuria, hand-foot syndrome, skin problems, headache, leukopenia, anemia, and diarrhea were not statistically significant compared with the control group (P > 0.05), indicating that the increase of anlotinib did not increase the risk of these adverse reactions. However, the incidence of nausea-vomiting [RR = 0.52, 95%CI (0.29, 0.92), P = 0.02] was lower in the anlotinib group, and the difference was statistically significant (P < 0.05). The study of Long Zuoyao et al. (2019) showed that if the patients have high basal values of alanine aminotransferase/menopartate aminotransferase and low albumin, they are prone to liver function abnormalities, with the clinical manifestations of malaise, loss of appetite, and nausea, and liver function and abnormal adverse reactions should be closely monitored during the administration of the medication in this group of patients.

According to the GRADE assessment, the evidence levels for IPFS, OS, and DCR are of moderate certainty, but ORR, nausea-vomiting have been downgraded to low certainty. These results support the use of anlotinib as a standard treatment option for brain metastases in NSCLC, particularly for patients who: require delay of intracranial progression (IPFS benefit); seek survival prolongation (OS benefit); or require long-term disease stability (DCR benefit). Clinical decisions should be based on high-quality outcomes, while low-quality evidence still requires further validation in the future.

However, this study still has some limitations: (1) Since anlotinib is a domestically developed drug in China, it has not been widely used in clinical studies abroad, resulting in a limited number of retrieved literature, most of which are small-sample studies. The analysis of IPFS and OS in this study is based solely on three studies, with a relatively small combined sample size, which may introduce the following limitations——Firstly, insufficient statistical power which a small sample size reduces the ability to detect the true treatment effect, potentially increasing the risk of false negatives. Secondly, limited precision of effect estimates, while the 95% confidence intervals for IPFS (0.36–0.75) and OS (0.54–0.88) are statistically significant, their wide ranges suggest uncertainty regarding the specific magnitude of the treatment effect. Additionally, the small number of studies may limit the applicability of conclusions to different populations of non-small cell lung cancer brain metastases, as excluded cohorts may differ in baseline patient characteristics and treatment regimens. The results for IPFS and OS should be interpreted with caution, as they represent preliminary evidence rather than definitive conclusions. Large-scale, multicenter RCTs are needed to validate these survival benefits. (2) Some RCTs did not clearly describe the random sequence generation method (e.g., did not specify the use of random number tables or computer randomization), which may amplify the efficacy signal. For retrospective cohort studies, despite a high NOS score (5–9 points), the inherent confounding bias of retrospective designs cannot be completely eliminated. Even after adjusting for known confounding factors such as age and gender in statistical models, unmeasured variables (e.g., treatment adherence, concomitant use of targeted therapy or immunotherapy) may still interfere, potentially overestimating the benefits of IPFS and OS by approximately 10%–15%. In clinical practice, decisions should be made by considering individual patient characteristics such as baseline risk and treatment accessibility. Future studies require large-scale, high-quality RCTs to conduct further sensitivity analyses and exclude the impact of bias on the results. (3) All the included studies were conducted in China, which may limit the generalizability of our research results to Western populations. Although there are no known significant racial differences in the expression or sensitivity of the targets inhibited by anlotinib, such as VEGFR, PDGFR, and FGFR; and the pathological and molecular characteristics of NSCLC brain metastases, such as tumor angiogenesis mechanisms and driver mutations, are consistent across different racial populations. But potential differences in drug metabolism and local treatment practices should be considered. Future multinational and multicenter trials should be conducted to evaluate the efficacy of anlotinib in Western NSCLC brain metastasis populations.

## 5 Conclusion

This systematic review and meta-analysis comprehensively evaluated the efficacy and safety of anlotinib in the treatment of brain metastases from NSCLC. The results showed that compared with the control group, anlotinib could significantly prolong the IPFS and OS of patients, and simultaneously increase the ORR and DCR. Anlotinib reduced the risk of nausea-vomiting, and did not increase the incidence of other adverse reactions. However, it should be noted that the quality of evidence in this study varies: OS and DCR are of moderate quality evidence, while IPFS, ORR, and nausea-vomiting are of low quality evidence. In summary, anlotinib can be used as a treatment option for patients with NSCLC brain metastases, providing survival benefits by prolonging IPFS and OS, enhancing the ORR and DCR to improve tumor control, and having the safety advantage of reducing the risk of nausea and vomiting. However, this study has limitations, including small sample sizes for IPFS and OS analysis, the study being mainly conducted on the Chinese population, and potential biases in some RCTs and retrospective cohort studies. Future large-scale, multi-center, and well-designed RCTs are needed to verify the above findings.

## Data Availability

The original contributions presented in the study are included in the article/Supplementary Material, further inquiries can be directed to the corresponding author.
